# WORKING LIFE EXPECTANCY AND UNPAID CAREGIVING: HOW WORKING LIVES DIFFER ACROSS CAREGIVER GROUPS

**DOI:** 10.1093/geroni/igad104.1049

**Published:** 2023-12-21

**Authors:** Lawrence Sacco, Brian Beach

**Affiliations:** Stockholm University, Stockholm, Stockholms Lan, Sweden; University College London, London, England, United Kingdom

## Abstract

Informal or unpaid caregiving (i.e. unpaid and untrained help and support to an ill relative or acquaintance) is becoming more prevalent as a result of population ageing and policies promoting ageing in place. Informal or unpaid caregiving is most common in mid-to-later life and can generate conflict with individuals’ participation in paid work at a time when working lives are being extended in several countries. Few studies have examined the impact of unpaid caregiving on working life as a whole. The current study examines how unpaid caregiving affects working life expectancy (WLE) among people aged 50 or older. Measuring paid work through WLE allows engagement in paid work in later life to be examined accounting for the fact that retirement transitions have become more complex due to the diversity of retirement pathways (e.g. un-retirement, phased retirement). Furthermore, using longitudinal data to measure WLE in the context of unpaid caregiving over several years enables us to differentiate among frequent caregivers and sporadic spells of caregiving. Data from the English Longitudinal Study of Ageing (ELSA) and the Swedish Longitudinal Occupation Survey of Health (SLOSH) were used to evaluate how WLEs are affected among individuals who provided unpaid care. We highlight the impact of different trajectories in unpaid caregiving on WLE, distinguishing caregivers according to intensity (hours and frequency per week) as well as provision of care over time.

